# The Optimisation of Carrier Selection in Dry Powder Inhaler Formulation and the Role of Surface Energetics

**DOI:** 10.3390/biomedicines10112707

**Published:** 2022-10-26

**Authors:** Olaitan Abiona, David Wyatt, Jasdip Koner, Afzal Mohammed

**Affiliations:** 1 Aston Pharmacy School, Aston University, Birmingham B4 7ET, UK; 2 Aston Particle Technologies Ltd., Aston Triangle, Birmingham B4 7ET, UK

**Keywords:** dry powder inhaler, surface energetics, lactose, design of experiments, aerosolisation, carrier morphology

## Abstract

This review examines the effects of particle properties on drug–carrier interactions in the preparation of a dry powder inhaler (DPI) formulation, including appropriate mixing technology. The interactive effects of carrier properties on DPI formulation performance make it difficult to establish a direct cause-and-effect relationship between any one carrier property and its effect on the performance of a DPI formulation. Alpha lactose monohydrate remains the most widely used carrier for DPI formulations. The physicochemical properties of α-lactose monohydrate particles, such as particle size, shape and solid form, are profoundly influenced by the method of production. Therefore, wide variations in these properties are inevitable. In this review, the role of surface energetics in the optimisation of dry powder inhaler formulations is considered in lactose carrier selection. Several useful lactose particle modification methods are discussed as well as the use of fine lactose and force control agents in formulation development. It is concluded that where these have been investigated, the empirical nature of the studies does not permit early formulation prediction of product performance, rather they only allow the evaluation of final formulation quality. The potential to leverage particle interaction dynamics through the use of an experimental design utilising quantifiable lactose particle properties and critical quality attributes, e.g., surface energetics, is explored, particularly with respect to when a Quality-by-Design approach has been used in optimisation.

## 1. Introduction

Dry powder inhaler formulations are designed to facilitate the delivery of powder-based therapeutic agents to the lungs. They are formulated as either loose clusters (carrier-free) comprising only the active pharmaceutical ingredient (API) or as a carrier-based formulation [1,2]. This review focuses on carrier-based DPI formulations, which generally comprise an interactive mixture of an API and a carrier, and is delivered through an appropriate passive inhalation device. In such carrier-based DPI formulations, since the carrier is often the main component, the aerosol performance of the formulation is largely dependent on the carrier characteristics such as particle size distribution, shape and surface properties. Therefore, carrier selection is crucial in determining the aerosolisation of the drug particles and their presentation for drug deposition in the lungs, due to interparticulate forces between the carrier and the respirable particles themselves [3]. Coarse carrier particles with mean particle size between 50 and 150 µm are used to improve the formulation, handling, flow and dispersion of the micronized API. During formulation, the drug particles attach to the surface of the carrier in an interactive mixture, but the API particles are expected to detach from the carrier upon aerosolisation and then be effectively dispersed to reach the deep region of the lungs [4]. Alpha lactose monohydrate is the most commonly used carrier in DPI formulation due to its ready availability, proven safety in inhalation products, its physicochemical properties, broad compatibility with most APIs, its stability and easy clearance from the body [5]. Despite the significance of carriers in DPI formulation performance, relatively little work has been reported on the use of having a readily measurable carrier particle property that will be useful in optimising and predicting DPI formulation performance through experimental design.

## 2. Lactose and Its Properties

Lactose is a disaccharide comprising galactose and glucose linked by a 1-4-glycosidic bond. It exists in both crystalline and amorphous forms. Crystalline lactose can be prepared either as Alpha-(α) lactose monohydrate (*O*-β-d-galactopyranosyl-(1-4)-α-d-glucopyranose) or anhydrous beta-(β) lactose (*O*-β-d-galactopyranosyl-(1-4)-β-d-glucopyranose), which contains about 70–80% anhydrous β-lactose and 20–30% anhydrous α-lactose depending on the temperature of crystallization [6].

Alpha lactose monohydrate is prepared by crystallizing a supersaturated solution of lactose at temperatures below 93.5 °C. The predominant crystalline shape depends on the conditions of crystallization, with prisms, pyramids and tomahawk shapes being the most common ones [7].

β-anhydrous lactose is a reducing sugar prepared by crystallizing at temperatures above 93.5 °C. Unlike α-lactose monohydrate, which is more thermodynamically stable, β-anhydrous lactose has a tendency for moisture uptake and reacts with primary and secondary amines when stored under conditions of high temperature and relative humidity. Due to the mutarotation of one anomer to the other, both α and β crystal forms of lactose coexist in equilibrium in solution regardless of the crystal form that is used in preparing the solution [6,7].

Amorphous lactose, which is the most reactive form of lactose, is produced when a solution of lactose is dried rapidly, such that crystallization cannot occur. It contains equal amounts of α and β lactose and is more readily reactive with primary and secondary amines than the crystalline forms [6,7].

### 2.1. Inhalation Grade Lactose

Inhalation grade lactose (IGL) could be either α-lactose monohydrate or anhydrous β-lactose. It is usually prepared from pharmaceutical-grade lactose under stricter manufacturing conditions for its specific application. Spray drying is also used to produce inhalation-grade lactose; spray-dried lactose contains up to 20% amorphous lactose. Although the amount of lactose ingested from a DPI formulation is safe in lactose-intolerant people, contamination with milk protein can cause anaphylactic reactions in those who have severe milk allergies. Hence, to eliminate the risk of anaphylaxis, IGL is subjected to broader microbiology specifications compared to other grades, such as endotoxin levels [5,6,8]

Different particle size fractions are generated by milling, sieving or air classification depending on specific requirements. These different production and processing methods coupled with batch-to-batch variation contribute to the history of the lactose particles. This history informs the lactose surface properties, most importantly its surface energetics, which influences particle–particle interactions with different APIs and ultimately affects DPI formulation performance. The size and morphology of α-lactose monohydrate crystals have been shown to vary based on the level of supersaturation of the solution used in the preparation [9]. In addition, several studies reveal the effects of batch and technological variations on lactose surface properties. Patera, Zamostny [10] studied the surface energies of α-lactose monohydrate, produced by milling and sieving and spray-dried amorphous lactose, both sourced from two different manufacturers. The results showed significant differences in surface energy for the same type of lactose from different manufacturers. Newell, Buckton [11] also studied the difference in surface energies of crystalline lactose, spray-dried lactose, milled lactose and a 99:1 physical mixture of crystalline and amorphous lactose. In addition to significant differences in surface energy values based on the grade of lactose, there was also a wide variation in the surface energy values of amorphous spray-dried lactose of the same batch, which was attributed to different surface orientations of the lactose molecules in the amorphous grade. In addition, Ticehurst, York [12] found that four batches of α-lactose monohydrate obtained from the same source exhibited different specific surface energy and different processing performance, which was attributed to variations in crystallinity or purity. Therefore, the presence of amorphous regions, surface impurities and the composite nature of the different lactose grades mean that there are continuous inconsistencies to handle with lactose carriers.

Furthermore, some studies show the resultant effects of variations in lactose properties on DPI formulation performance. In a study carried out by Steckel, Markefka [13], IGL was prepared from five different batches of the same grade of lactose (Lactochem^®^ crystals, DFE Pharma, Nijmegen, the Netherlands), which varied in mean diameter, and had agglomerate and fines content more than usually present in batch-to-batch variations. They found significant differences in the efficacy of DPI formulations produced using IGL obtained from the different batches of Lactochem^®^, especially in DPI formulation blends containing low API concentration. Likewise, Larhrib, Zeng [14] examined the effects of using five different lactose grades with similar VMD and found substantial differences in the efficiency of delivery of salbutamol sulphate from DPI formulations prepared using the different lactose carriers. In addition, Pitchayajittipong, Price [15] report an increase in fine particle dose as the fluidisation energy of the lactose carrier used increases. Therefore, the preparation history determines the surface properties of the lactose particles and, most importantly, the surface energetics, which influences particle–particle interactions between the carrier particles themselves and any respirable API particles, ultimately affecting the performance of the DPI formulation. Despite the significance of carriers in DPI formulation performance, relatively little work has been reported on the use of having a readily measurable carrier particle property that will be useful in optimising and predicting DPI formulation performance through experimental design.

### 2.2. Challenges Associated with Carrier (Lactose)-Based DPI Formulations

Micronized APIs are notoriously difficult to handle due to their cohesive force, which is usually greater than the dispersion force generated during aerosolisation [16]. For this reason, the use of carriers generally in DPI formulation eases the pharmaceutical handling, formulation and production of the micro fine particles of the active materials. The use of carriers is however not a panacea. Indeed, DPI formulation carriers affect the delivery of APIs to the lungs, as incomplete detachment from carrier particles leads to sub-optimal delivery of administered drugs [2]. It is also important to have a balance between the drug–carrier adhesion and the force needed for deaggregation, such that the API–carrier system does not separate during handling, dispensing and transportation, and still allows optimal separation of API from the carrier during aerosolisation [17].

Complete detachment of the drug from the carrier, which is difficult to achieve, is required to maximise the formulation performance. Ultimately, the detachment of API is dependent on the cohesive–adhesive balance of the drug–carrier mixture, which determines the strength of the interactive mixture and ease of drug detachment from the carrier when required [18,19,20,21].

The predominant physical forces on particle surface are Van der Waals interaction, the electrostatic force from charge separation, capillary forces determined primarily by environmental moisture and forces associated with mechanical interlocking. These forces control the interaction between the drug and carrier particles. Van der Waals interaction is a result of transient dipolar attraction between the surfaces of the particles when they are in close proximity to each other. Capillary forces occur due to the formation of condensed vapour at the interface of two lyophilic particles that are in contact with each other; electrostatic attractive interactions occur when two oppositely charged particles encounter each other, while mechanical interlocking can result from lock and key conformation between particles due to surface irregularities [4,22].

Since the interaction between drug particles and lactose particles is based on surface forces, the physicochemical and surface properties of lactose are capable of influencing this interaction. For example, the conditions of manufacturing processes such as the temperature of crystallization, absolute concentration of supersaturated lactose solution, presence of impurities, nucleation event(s) and cooling profile all influence the size, shape, and surface amorphous content and the energy distribution of the lactose carrier [4,23]. The rugosity (surface roughness or surface smoothness) and shape of lactose particles influence the surface area of contact for particle interaction and the strength of adhesion force between the drug and carrier particles. Similarly, the surface characteristics of the lactose carrier used will also affect compatibility with API and the stability of the DPI formulation [4,24,25]. The active pharmaceutical ingredients delivered by DPIs are often highly potent and must be delivered in very low quantities; hence, the carrier makes up the bulk of the formulation. The carrier properties are therefore critical to the overall DPI formulation performance, and any change in carrier properties will have a pronounced effect on the delivery of the API [3,26]. Due to these variations, with the resultant differing physicochemical properties of the carrier, for guided carrier selection and optimal DPI performance, it has been important to study the effects of different carrier properties on DPI performance. Specific lactose properties such as size, shape, and surface morphology have been investigated extensively, and their effects on DPI formulation performance are summarised in Section 4. However, the interactive effects of carrier properties on DPI formulation performance make it difficult to establish a direct cause-and-effect relationship between a single carrier property and its effect on the performance of a DPI formulation. Alpha lactose monohydrate remains the most widely used carrier for DPI formulations. The physicochemical properties of α-lactose monohydrate particles, such as particle size, shape and solid form, are profoundly influenced by the method of production. Therefore, wide variations in these properties are inevitable. In this review, the role of surface energetics in the optimisation of dry powder inhaler formulations is considered in lactose carrier selection. Several useful lactose particle modification methods are discussed as well as the use of fine lactose and force control agents in DPI formulation development. It is concluded that where these have been investigated, the empirical nature of the studies does not permit early formulation prediction of product performance, but rather only allows the evaluation of the final formulation quality. The potential to leverage particle interaction dynamics through the use of an experimental design utilising quantifiable lactose particle properties and critical quality attributes is explored, particularly with respect to when a Quality-by-Design (QbD) approach has been used in the optimisation.

## 3. Mechanism of API Detachment from Carrier

Given the difficulty of studying the effect of lactose physicochemical properties in isolation, it is important to consider how several factors may simultaneously affect API aerosolisation. There are two major mechanisms by which API particles are believed to detach from carrier particles, viz, detachment by airflow stream or fluid forces, and detachment by transfer of momentum caused by impaction when carrier particles collide with the inhaler or capsule wall [27].

An airflow stream is generated when the powder bed is aerosolised upon inhalation. For the detachment of API particles to occur through this mechanism, it is essential that the carrier surface has minimal asperities, which allows for a clear path for the airflow to access and remove the API particles [27,28].

Mechanical forces, on the other hand, are generated when carrier particles impact the inhaler wall, leading to momentum transfer from the carrier to API particles, which then generates detachment force. The detachment force generated is proportional to the amount of momentum transferred, which is, in turn, dependent on the mass of the carrier particle, assuming a constant velocity of the different carrier sizes in the formulation [27].

Although detachment by mechanical forces may not be obstructed by surface asperities as much as detachment by flow, the detachment force generated must be greater than the adhesive force between API and the carrier, and the position of the particle must favour removal of the API particles [27,29].

Fine API particles attached to fine carriers are more likely to be detached by the airflow stream generated during aerosolisation than API particles attached to coarse carriers. API particles on larger carriers are more likely to be detached by mechanical forces due to impaction. This difference is due to a combination effect of carrier size and surface morphology. Smaller carriers have smoother surfaces from which APIs can be easily detached, since the API particles are freely exposed to the airflow stream. As carrier size increases, there are correspondingly more asperities where drug particles can be shielded from airflow during aerosolisation of the powder bed. This results in the likelihood of the API being aerosolised through detachment via mechanical force and momentum transfer. Additionally, since momentum transfer is directly proportional to the mass of the carrier particle, larger carriers will transfer more momentum required for drug particle detachment [27,29]. It is important to note that this may result in the deposition of the drug in the inhaler/delivery device (undelivered dose) if the position of the particle is not favourable. This was the case in a study by Steckel and Müller [30] in which undelivered doses from an interactive mixture of budesonide and α-lactose monohydrate of different sieve fractions increased as the carrier particle size increased.

Additionally, as API particle sizes decrease, the force required for detachment increases, the same applies to API particles attached to high-energy sites, due to higher adhesive forces between API and carrier particles. While larger API particles, either fine drug agglomerates or large primary particles, have a higher surface area and better aerodynamic profile to interact with the airflow stream and be detached through the airflow stream, smaller primary drug particles require greater detachment force usually by mechanical detachment. This influences the respirable fraction of the formulation, with detachment by momentum transfer generating more primary particles than detachment by airflow stream, in which, predominantly, either aggregates or larger primary particles are detached [27]. This was evident in a study by Kaialy, Alhalaweh [31], in which, although lactose carrier particles with overall smaller VMD increased the fine particle fraction (FPF), there was a concurrent increase in deposition of budesonide in the throat region, which increases the potential for side effects.

## 4. Carrier Factors Affecting Performance of DPI Formulations

### 4.1. Effect of Carrier Particle Size

The consensus on the effect of carrier particle size on drug aerosolisation is that smaller carriers are more favourable for drug dispersion [30,31,32,33]. However, it is difficult to examine the effects of individual physicochemical properties of the carrier on DPI performance in isolation. This is because of the interdependence of physicochemical properties, as such, changing one property, e.g., particle size, may generate other variable(s), e.g., particle shape, surface roughness, surface area or surface property. Therefore, several variables may be present while only one is being examined. In order to overcome this challenge, Ooi, Traini [34] used a series of model polystyrene spheres to examine how carrier size, independent of other variables as much as possible, affects aerosol performance. They investigated the aerosolisation of salbutamol sulphate from model polystyrene carrier particles of three different particle sizes. These model carrier particles were deliberately prepared to have a similar density, shape, surface roughness and chemistry, thereby reducing the number of between observations. This study shows that, in isolation from other variables, as carrier size decreases, a concurrent increase in drug aerosolisation performance occurs. The significant increase in aerosol performance was primarily attributed to more frictional and rotational collisions between particles of the smaller carrier in the powder bed, facilitating API detachment from the carrier during aerosolisation. Although this model carrier is not typical of an ideal drug–carrier system, it provides useful data to evaluate potential similarities or differences in ideal drug–carrier systems where multiple variables may be present simultaneously [34].

Earlier research to investigate the effect of lactose size on in vitro deposition of salmeterol xinafoate reported similar findings as above, where FPF increased with low carrier particle size <32 µm [30]. Ideally, as particle size increases, surface area and surface energy decrease. It is expected that this should reduce drug–carrier adhesion and facilitate drug dispersion. However, the opposite trends observed were attributed to the higher impact that larger carriers have on the inhaler wall during aerosolisation of the powder bed, which results in more undelivered doses. This is aggravated by the fixed drug:carrier mass ratio, which results in a higher drug-to-carrier surface area ratio with larger carriers, in which there will be more drug particles per carrier with the use of larger carriers, since the number of carrier particles reduces with larger carriers [30,34]. This observation is consistent with that of [34], where one of the other factors thought to facilitate drug dispersion from smaller carriers is the lower drug: carrier surface area ratio along with the increased number of carrier particles, which promotes more frictional and rotational collisions between particles. These explanations link drug dispersion directly to the innate volume mean diameter (VMD) of the carrier, which is not the case in more recent studies.

More recently, studies have attributed the improved dispersion observed with smaller lactose carriers to the fine content rather than inherent particle size. This is a more plausible explanation considering that the presence of fine and coarse carrier fractions will favour the two main mechanisms of drug detachment described in Section 3. Islam, Stewart [33] investigated the direct effect of six different grades of lactose on the dispersion behaviour of salmeterol xinafoate (SX). They found that as the VMD of lactose decreased, the FPF of SX increased. They speculated that this could either be due to the distinct ability of decreasing lactose particle size to improve dispersion, or a function of the fine lactose content in the carrier, which increases as VMD decreases. The removal of fine lactose by wet decantation from two of the six lactose grades used in this study and the subsequent decline in SX dispersion supported the latter claim [33]. Similarly, in a study by Guenette, Barrett [35], different size fractions of lactose were mixed in different proportions based on experimental design to produce ten lactose carrier blends with D_50_ between 29.74 and 89.54 µm. These lactose blends were used to investigate the aerosolisation performance of salbutamol sulphate (SS). The formulations containing the highest amount of ultrafine (<10 µm) and fine lactose (10–40 µm) produced the highest FPF, this was also attributed to the fine lactose content. Similar observations have been found in other studies; although, the fine lactose content desirable in improving dispersion needs to be balanced to avoid undesirable effects such as poorer flow, less homogeneity and increased possibility of carrier deposition in lower airways [30,31]. Other recent studies have directly investigated the effect of extrinsic lactose fines and processing times on the aerosolisation behaviour of DPIs containing a model drug [36]. This is considered later on in Section 6, as a means of lactose surface modification rather than as an effect solely down to carrier particle size, due to the other different factors (such as change in surface roughness, surface energy, and potential reduction in high energy sites) that come into play to impact drug aerosolisation.

### 4.2. Effect of Carrier Particle Shape and Surface Morphology

Particle shape and morphology can be characterised using several descriptors, which are the elongation ratio (ER), flatness ratio (FR), roundness (RO), shape factor (F_shape_) and surface factor (F_surface_). ER and FR are the basic parameters, hence, referred to as first-order descriptors. In simple terms, ER is the ratio of particle length to width, while FR is the ratio of width to thickness. With reference to perfect cubes and spheres, which have ER and FR of one, more elongated particles have ER > 1 while flatter particles have FR > 1 [31,37]. RO is a measure of both particle geometric shape and surface smoothness, a sphere with a smooth surface has a roundness value of one, while a sphere with a rough surface and the other shapes have a roundness value greater than one. Therefore, the higher the roundness value, the more irregularly shaped and/or the rougher the surface of the particle is (14). F_shape_ is a second-order descriptor, given that it is a measure of particle shape irregularity based on particle orientation and contact area, but independent of actual particle dimensions. F_shape_ values range from −1 to 1, with more irregularly shaped particles having smaller values. While ER, FR, RO and F_shape_ define particle shape, RO and F_surface_ characterise particle surface texture or morphology. Thus, F_surface_ is referred to as a third-order descriptor. Regular particles with smooth surfaces have an F_surface_ value of one, while a decrease in the F_surface_ value indicates rougher surfaces [38].

Kaialy, Alhalaweh [39], using ER (values between 1.62 ± 0.04 and 5.89 ± 0.2) and roundness (values between 1.36 ± 0.13 and 6.49 ± 2.91) as the descriptor of carrier shape, investigated the effect of five different carrier shapes on salbutamol sulphate. Although they discovered that the higher the ER of the carrier particle, the higher the deposition of salbutamol sulphate to the lower airway regions, it is important to note that there were significant variations in other physicochemical properties. To support the estimations obtained by shape factor descriptors, scanning electron microscopy (SEM) micrographs and particle size distribution revealed the extent of the variation in particle size, shape, surface morphology, degree of surface roughness/smoothness, surface asperities and even the amount of intrinsic carrier fines, while the effect of particle shape was the only focus of this study. It is interesting to note that carrier particles with a higher ER (acetone-crystallised mannitol ER = 4.83 ± 0.18 and ethanol-crystallised mannitol ER = 5.89 ± 0.19) were the same ones with a smoother surface, they also had a smaller VMD and a higher number of intrinsic fines up to 8.6%. These observations further highlight the difficulty of investigating one physicochemical property in isolation, as the additional factors that were not in consideration may well have influenced the overall conclusions.

With respect to lactose as a carrier, Kaialy, Ticehurst [38] carried out a comprehensive study, which acknowledged the consolidation of multiple lactose shape components on DPI performance. In contrast to the above study, variation in particle size was eliminated. All lactose carriers used had similar VMD between 81.6 ± 2.6 and 84.9 ± 1.3 µm, and the same amount of fine lactose, less than 1% of fine (<10 µm) lactose fraction. As in the above study, the lactose carrier with the highest ER deposited the highest amount of salbutamol sulphate to the lower airway regions. However, the better aerodynamic performance was attributed to the co-effects of the high surface roughness and shape irregularity of the lactose carrier. A recent investigation has shown the fabrication of slab-shaped lactose carrier particles through air jet milling, which significantly impacts the shape and surface roughness [40].

Another comprehensive study by Larhrib, Zeng [14] emphasises the co-effects of different lactose carrier physicochemical properties. Five different grades of lactose carrier were investigated for their effects on DPI performance, in which ER and roundness were the only quantitative descriptors of lactose carrier shape. Interestingly, the fine particle dose and FPF more than doubled significantly when an amorphous lactose grade was used, which, in addition, had the lowest ER. In addition to considerable particle size variations, several variations revealed by SEM micrographs, such as surface roughness and asperities, were higher in the crystalline lactose grade. Amorphous lactose had a considerably higher amount of fine lactose content (23% < 10 µm and 12% < 5 µm), which was the main reason given for its efficient delivery of salbutamol sulphate.

In addition, using different grades of lactose carriers, Kho and Hadinoto [41] investigated the effect of three different lactose carrier shapes (tomahawk—CL, needle—NL and pollen shaped—PL) at two differing size fractions, i.e., 50–70 µm and 14–20 µm, on the delivery of amorphous ciprofloxacin nanoparticles. The aerosolisation efficiency of the API–carrier particle blends was compared with that of the carrier-free inhalable aggregates of ciprofloxacin nanoparticles. For the smaller lactose size fraction (14–20 µm), they reported no significant increase in emitted dose and FPF using the NL lactose carrier compared to the carrier-free formulation. This was despite NL having the highest ER of 4.1 compared to 1.5 and 1 for CL and PL, respectively. This is likely due to the poorer flow linked to formulations with carriers that have a high ER, as well as the higher potential for drug loss and drug deposition in the throat region, which reduces the emitted dose and FPF [41]. In contrast to NL, CL and PL lactose were able to double the FPF to 17 ± 1% and 16±1%, respectively, from 8 ± 2% for the carrier-free formulation. Although, the mass median aerodynamic diameter (MMAD) for all formulations was similar to that of the carrier-free formulation (between 5.5 and 6.0 µm), suggesting that the drug particles were deposited as aggregates of ciprofloxacin nanoparticles. This was confirmed by the NGI deposition pattern of the ciprofloxacin nanoparticles, which were mainly deposited in stage 1, with an average aggregate size greater than 6 µm. The drug detachment mechanism supports this observation, since the small carrier size used favours API detachment by the airflow stream, which is more likely to release large drug particles or fine drug agglomerates [27].

For the larger CL, NL and PL (50–70 µm), all three were able to increase the emitted dose to 70–80% from 52% for the carrier-free formulation; this was attributed to the ability of larger carriers to reduce drug aggregates. This increase in emitted dose did not produce a corresponding increase in FPF for the formulation prepared with PL, which remained at 8 ± 2%. This was attributed to poor drug detachment from the crevices on the PL carrier surface. Rationally, undetached API from the carrier surface does not negatively affect the emitted dose, as it potentially deposits in the earlier stages of the NGI with the carrier particles, which also contributes to the emitted dose but not to FPF. The NL and CL carriers, however, increased FPF to 17 ± 2% and had similar deposition patterns. Overall, this study showed that there was no superiority in carrier particle performance despite the difference in shape [41].

## 5. The Mixing Process

Fan, Chen [42] describe solid mixing as the process of randomly dispersing two or more particulate solids amongst each other by the random movement of the particle. The primary purpose of solid mixing is to achieve blend homogeneity; the components of a homogenous mixture are uniform throughout the whole blend, and random samples, which are expected to be representative of the whole mixture, are analysed to determine blend homogeneity [42,43]. The mixing process of powders used in DPIs and other pharmaceutical formulations is vital to the quality and performance of the product. The need for blend homogeneity in DPI blends cannot be over-emphasised, as this directly affects drug content uniformity, the lack of which may result in overdosing of API, dangerous side effects and potential death, and the underdosing of API, which may result in poor therapeutic effect and deterioration of patient disease and health.

Despite being an age-old process, powder mixing still very much lacks a uniform scientific approach, and is commonly treated as an art based on previous experience and trial and error. In addition, the wide range of particle size, size distribution, shape, density and chemical composition of materials in DPI formulation creates a barrier to process development, as there is no one size fits all. For instance, the challenges posed by free-flowing materials (segregation) differ from those encountered when mixing fine or poor-flowing materials (agglomeration) [43]. It is critical to understand the nature of the materials to be mixed, and the mixing mechanisms of different mixers/blenders, in order to select the most appropriate type of blender to achieve a homogenous mixture [42]. The three major mixing mechanisms are described as follows.

### 5.1. Mixing Mechanisms

#### 5.1.1. Convection

This describes the movement of a group of particles within the powder bed from one part of the mixing vessel to another. This movement is usually as a result of a rotating blade or paddle. As the impeller moves, it moves the particles alongside, increasing surface contact between the particles, and homogenising the mixture [42,43].

#### 5.1.2. Diffusion

In diffusion, the movement of the impeller causes movement of individual particles relative to one another. Over time, there is a reshuffling between the particles as they are spread over new surfaces as a result of their random motion. This causes new particle–particle interactions to occur, which facilitate homogenous mixing [42,43].

#### 5.1.3. Shear

In convective mixing, narrow zones of high velocity exist between groups of particles referred to as slip zones. The high velocity coupled with the narrow size of these slip zones allows shearing to occur. As agglomerated particles move through these zones, they are forcefully compressed against mixing vessel walls and broken up; hence, this mechanism is also reported to cause the deagglomeration of cohesive powder [43,44].

An understanding of these mixing mechanisms helps when selecting the mixer for a specific purpose. For example, in DPI formulations, where fine APIs are mixed with large carrier particles, the deagglomeration of fines (API) is critical to the process. Therefore, it will be important to select a mixer that is able to break up agglomerates [17]. While mixers that predominantly operate by convective and shear mechanisms work better to break up agglomerates, those that operate predominantly by diffusion require longer mixing times to do the same. Increased mixing times, however, can increase the potential for unintended segregation, i.e., demixing, and be detrimental to blend homogeneity [45]. However, the increased particle–particle interaction created during diffusive mixing is highly favourable in achieving a uniform drug–carrier blend caused by particle redispersion. Apart from the desired blend quality, there are other factors that need to be considered in designing a mixing process, such as the drug:carrier ratio in terms of size and quantity, batch size, sensitivity of materials to heat or moisture, adhesiveness of materials onto mixer surfaces, potential for contamination, potential for unwanted particle size reduction and triboelectrification [43].

The most commonly used mixers in DPIs are low-shear mixers such as TURBULA^®^ mixers and high-shear mixers such as the Turbo Rapid Variable (TRV) Mixer. Low-shear mixers have been predominantly developed for powder mixing, whereas high-shear blenders have a heritage of mixing liquids and solids. High shear mixers utilise all three mixing mechanisms of operation (diffusion, shear and convection), which ought to deliver the best conditions for the deagglomeration of fine API and subsequent homogeneous dispersion of the API on the carrier particles [46]. However, with DPI formulations, beyond creating a good quality homogeneous mixture, which is able to withstand agitation during handling, packaging, transportation, storage, etc., up until the point of use, it is critical to creating a balance between drug–carrier adhesion and subsequent drug detachment, which is essential for API delivery to the lungs. The drug–carrier adhesive force determines the formulation homogeneity and ability to withstand agitation, while this adhesive force needs to be overcome for optimal DPI formulation dispersion and performance. Recent developments in mixing technology, specifically isothermal dry particle coating (iDPC), hold out the prospect of predictive and science-driven mixing and coating rather than black art [46,47].

## 6. Optimisation of DPI Formulation Dispersion by Carrier Surface Modification

Different methods have been explored to modify carrier surface properties in order to control DPI dispersion behaviours. These methods have been reviewed extensively by different authors and are summarised in Table 1.

### 6.1. Addition of Fine Lactose Particles

The incorporation of fine lactose particles in a carrier-based DPI formulation has been investigated considerably. Supported by two major theories, it is established that the inclusion of lactose fines enhances formulation performance [26,58,59,60]. The first is the “active site” theory, which explains that fine lactose particles attach to potential drug binding sites, leaving the drug particles to attach to less active sites on the coarse lactose carrier surface from which they are more easily detached on inhalation. Surface active sites on lactose carrier particles have been described based on surface morphology, rugosity, irregularity, presence of impurities, surface energy and chemical properties [61]. Surface crevices, which are larger than drug particles and reduce the tendency for drug detachment are presumed to be active sites. There are also sites with higher surface energy, related to polar or dispersive forces, increased surface interaction forces, e.g., van der Waals or capillary forces; and sites that are charged or chemically contaminated [51,62]. There is, however, no evidence of preferential binding of lactose fines over drug particles or vice versa at such “active sites”; thus, a lactose fines before drug particles blending order is important for improved DPI formulation performance based on this theory [22,26].

The second is the “agglomerate theory”, which was put forward based on contrasting evidence where blending order did not influence the formulation performance [63,64]. This theory states that the dominant mechanism affecting attachment is that fine lactose particles preferentially form agglomerates with the fine drug particles rather than simply blocking active sites on coarse lactose resulting in co-deposition and enhanced aerosolisation of the API [65]. Upon inhalation, the fine drug particles are more easily detached from the fine lactose surface due to the smoother carrier surface, or the carrier–drug agglomerates may be small enough to be delivered as part of the respirable dose. There is also the possibility of easier detachment of the drug–carrier agglomerates, since the greater mass of the aggregated particles allows for a greater effect of fluid forces for drug detachment by the airflow stream [20,48,64,66]. The mechanisms of drug detachment from carrier particles as described in Section 3 provide further support to the agglomerate theory.

### 6.2. Use of Force Control Agents

To mask the “active sites” on coarse lactose, force control agents (FCAs), which are generally materials thought to reduce cohesion and adhesion have also been used to modify the carrier surface and facilitate dispersion. The most commonly used are magnesium stearate and L-leucine, since the unproven toxicological profile of potential anti-adherents restricts the choice of material for delivery to the lung [51]. Indeed, a recent study has explored the mechanistic behaviour of magnesium stearate on the aerosolisation pattern of model APIs [67]. Some studies have also explored polymers as potential surface-coating agents for lactose [24].

Ref. [68] investigated the effect of coating three different lactose carriers of mean diameter 10 µm, 60 µm and 150 µm with magnesium stearate and sucrose stearate, on the surface properties of the carriers, and how this impacts the aerosolisation behaviour of a particular but unnamed drug. Generally, they found that formulations containing lactose coated with magnesium stearate (MgSt) and sucrose stearate deliver higher FPF with a correspondingly increased deposition of the drug in the lower stages of an Anderson Cascade Impactor (ACI), when compared to the performance of formulations of an uncoated carrier. This was attributed to the ability of the FCAs to reduce adhesion between the drug and lactose carrier. This was also supported by the fact that more of the drug dose remained undelivered due to retention in the inhaler device, which was attributed to reduced drug–carrier adhesion. There was also a dose-independent increase in FPF with magnesium stearate and sucrose stearate, which was due to weak drug–carrier adhesion, even at high-energy binding sites, because of the force-controlling effects. Likewise, a recent study carried out by Benke, Farkas [69], compares the aerodynamic properties of micronized meloxicam potassium in a carrier-free formulation, lactose-based formulation and surface-modified lactose (with MgSt) carrier-based formulation, with spray-dried meloxicam potassium in the three formulations. The authors report better lung deposition for the surface-modified lactose carrier-based formulations in the two categories of micronized and spray-dried meloxicam potassium.

There is also the potential to use FCAs in carrier-free formulations, since they primarily reduce interparticulate forces, whether adhesive or cohesive as would be the case in a pure drug formulation. Begat, Morton [70] investigated the effects of MgSt, leucine and lecithin on the performance of salbutamol sulphate and budesonide in a carrier-free system. All three FCAs produced a significant increase in FPF and a corresponding reduction in the MMAD of the aerosolised drug. These authors suggested that this was due to the FCAs’ ability to reduce interfacial free energy between the drug particles, and hence reduce cohesion and facilitate deagglomeration.

Other methods such as carrier surface smoothing have also been used to facilitate DPI aerosolisation, by evening out crevices where drug particles would otherwise be trapped as highlighted in Table 2. However, contrasting evidence shows that carrier surface roughening likewise enhanced DPI performance. It is suggested that these conflicting data are the result of diversity in terms of blending equipment, inhaler device, nature of drug particles, etc., which complicate the direct cohesive–adhesive interaction between lactose surface morphology and DPI performance [51].

## 7. Surface Energetics and Particle Interactions

To determine the performance of DPI formulations, FPF is used as a measure of the aerodynamic dispersion of bulk powder into primary particles or drug agglomerates. For dispersion to occur, separating forces have to overcome interparticulate attraction within the bulk powder to produce primary particles of API in the respirable size range. Therefore, overcoming interparticulate interactions is critical to DPI performance [4]. Despite an understanding of the interparticulate interactions involved in particle adhesion and cohesion and the study of particle properties and how they affect DPI performance, the effects of bulk powder properties on DPI performance are still not fully understood and the correlations between the two have not allowed consistent prediction of DPI formulation performance. This is due to the complexity created by the interactive influence of particle properties such as size, shape and surface morphology on DPI formulation performance making it difficult to predict DPI performance based on particle properties alone. Therefore, it is highly recommended to establish a relationship between the comprehensive powder properties of DPI formulations and pulmonary drug deposition for the purpose of DPI formulation performance prediction, to accelerate the process of research and development in early formulations screening, based on powder properties [50].

Surface energy, an intrinsic particle property has the potential to establish such a relationship and to be used as a predictive tool for DPI formulation performance [76,77]. Surface energy refers to the free or excess energy at the surface of a solid particle compared to the bulk. Solid particles consist of molecules, which, within the bulk, are normally bound together on each side. However, at the solid–gas interface, due to the intermolecular attraction towards the bulk of the particle, there is a net force on the molecules away from the particle surface, leaving unbound atoms at the surface. The measurement of this excess energy at the solid surface, due to the unbound atoms, is known as surface energy [78]. There are two components of surface energy, dispersive and polar surface energy. Dispersive surface energy is a result of fluctuations in charge distributions, which induces transient dipoles, i.e., van der Waals interactions. Polar surface energy is due to permanent dipoles from charged particles or polar groups on the particle surface. Since surface energy is a result of intermolecular attractions within the particle, there is a direct relationship between the strength of intermolecular attraction, surface area and surface energy. Therefore, the stronger the intermolecular attraction, the higher the surface energy, and as surface area increases, more molecules are forced to the particle surface, hence increasing surface energy [79]. Interactive mixtures are formed using the thermodynamic instability created by unbound atoms at the solid particle surface, which allows for the physical adsorption of molecules to the particle surface. ‘Solids with high surface energy have a high tendency to form strong bonds with other materials and vice versa. The stronger the bond between the drug and carrier particles in an interactive mixture, the less easy it is for the drug to detach. Correspondingly, the weaker the bond, the less stable the interactive mixture and the more easily particles can be detached from the carrier [78,80]. The relationship between surface energy, interparticle bond strength and formation of interactive mixtures may be leveraged as a predictive tool in designing the performance of DPI formulations. For this reason, the measurement of surface energy has become an important part of formulation development.

### 7.1. Surface Energy Determination

There are a number of ways to measure surface energy including interaction with water vapour [81], by contact angle measurement [82,83], atomic force microscopy (AFM) [84,85,86] and most recently, inverse gas chromatography (IGC) [87,88]. The use of contact angle and AFM methods in surface energy determination is limited by challenges such as the reactivity of particulate pharmaceutical materials to contact angle fluids and ambiguous indices for AFM [89]. The IGC method is particularly advantageous primarily because it allows materials to be characterized in their native state thereby retaining the integral particle surface properties more representative of those in the formulation. IGC requires minimal sample preparation, and samples can be recovered post analysis, i.e., it is a non-destructive method.

### 7.2. Use of IGC in Measuring Surface Energetics

The basic concept of IGC is that of gas chromatography (GC). In contrast to conventional GC, IGC measures the unknown surface of a sample powder that is packed in a column into which a series of known alkane probe vapours is consecutively injected. There are two types of IGC, IGC at infinite dilution (IGC-ID) occurs at low and fixed probe concentrations, usually less than 0.01 P/P_0_. In this case, the probe molecules act independently and due to the low concentration of probe molecules, there is an increased tendency of preferential interaction with high-energy sites. Therefore, the surface energy values obtained from IGC-ID are more representative of the highest energy sites [87,90]. Secondly is IGC at a finite concentration (IGC-FD), in which case, different probe concentrations are injected into the sample column to target a fractional surface coverage of the material, usually between 0% and 20%. This allows more interaction between the probe molecules with different energy sites and gives a surface energy heterogeneity profile of the material, which is more representative of the total energy sites. The surface energy of the powder is calculated from the retention times of the probes. The acid–base properties of the particle surface are determined using monopolar basic and acidic probes [91,92].

### 7.3. Use of Surface Energetics to Optimise and Control DPI Performance

Many different methods have been investigated in the creation of engineered particles to control adhesion/cohesion forces both between micronized drug and carrier particles and also between the micro-fine drug particles themselves. Methods such as particle surface smoothening, roughening, and coating the carrier surface with force control agents have all been found to influence DPI formulation performance. There remains a gap between formulation design and converting theory to practice by using such materials to control the manufacturing process to deliver DPI formulations with the targeted aerodynamic performance. These particle-engineering methods will no doubt have effects on the surface energetics of the particles, since particle history and the presence of surface contaminants are major factors that influence surface energy. The importance of particle surface energetics in determining particle adhesion and cohesion means that surface energy could potentially be used in the manufacturing process to predict and optimise DPI formulations through a quality-by-design approach. This would not only accelerate product development but also give regulators more confidence in the formulations being processed. Therefore, in addition to using other physicochemical properties to optimise DPI performance, particle surface energetics could be potentially used to both optimise and predict DPI formulation performance.

The challenge with solid surface energetics is the heterogeneous nature of the powder surface. Unlike the liquid state where the free movement of molecules homogenises surface energy, surface energy is unevenly distributed and heterogeneous, with some regions possessing higher surface energy than others. It is more realistic to characterise a surface with a range of surface energy values over a specified surface area, but a median value is often specified to represent the overall surface. In reality, adhesion characteristics differ within the same batch of powder [93].

The complexity associated with different surface energy values and their effects have been reported in several studies. Two different studies by Kumon, Suzuki [68], Das, Zhou [80] showed that surface energy values obtained at infinite dilution mainly reflect that of high energy sites, whereas the supposed passivation of the lactose particle surface with MgSt increased rather than decreased the surface energy, as would have been expected. API aerodynamic performance was reported to be better despite the increased surface energy.

Another study by Cline and Dalby [77] aimed to correlate surface energy values of carrier particles with the FPF of an interactive mixture whilst incorporating the specific surface area to understand the amount of surface available for interaction. This study shows a similar correlation between FPF and surface energy interaction to the studies mentioned above in which only surface energy was correlated with FPF. The unexpected increase in FPF as surface energy interaction increased is believed to be due to the fact that a minimum threshold of surface energy interaction between drug and carrier particles is required to facilitate better dispersion of cohesive drug particles and increase aerodynamic performance. Below this threshold, drug particles may remain aggregated and produce low FPF [77]. In addition to median surface energy values and energy distribution, a wider set of data can be obtained from the IGC measurement of surface energy. Work of cohesion and work of adhesion, which represent the thermodynamic interaction between similar and dissimilar surfaces respectively can be calculated. Using these values, a cohesion–adhesion balance, which is the ratio of work of adhesion to work of cohesion, can be obtained. Interestingly, a review of studies in which the in vitro inhalation performance of DPI formulations is correlated with the cohesive–adhesive balance identified, and also shows a similar trend as those mentioned earlier. Ref. [20] investigated 16 drug–carrier formulations; optimal performance was reported in those with a slightly cohesive cohesion–adhesion balance. Another study by [19] investigated the correlation between the cohesion–adhesion balance of salbutamol sulphate with different carriers and FPF; they reported similar findings, a corresponding increase in FPF with an increase in the cohesion–adhesion balance. These findings all agree that a level of drug–carrier adhesion has to exist for optimal DPI performance. However, there is a need to control this API–carrier adhesion strength to enable optimal separation of API from carrier particles when required.

The investigation of surface energy and its derivatives provides useful information about DPI formulations, demonstrating that both cohesive forces between micronized API particles and adhesive forces between API and carrier particles are critical to aerodynamic performance. While the use of surface energy, surface energy interactions and the cohesion–adhesion balance (CAB) approach cannot precisely replicate the exact interactions between drug and carrier particles in a formulation; the ability to quantify surface interactions between drug and carrier may provide a useful structure to predict and optimise the possible behaviour and performance of a carrier-based DPI formulation. The quantitative representation of surface interactions eliminates the subjectiveness associated with qualitative examination, and mathematical and statistical models can be used to predict and optimise DPI formulation performance. Correlations obtained in this way may accommodate other particle properties such as size, shape, morphology, polymorphic form, presence of impurities, etc., as these factors all contribute to the surface energetics of the particle. Despite the promising prospects of using surface energy for formulation development, it is unlikely that a one-size-fits-all approach will be found. This is because surface energy values are specific to materials; therefore, significant changes in formulation behaviour may be expected with different carriers and APIs as a function of their differing cohesive–adhesive properties. Considering that lactose is the most commonly used carrier, a larger proportion of research should be dedicated to lactose. Even for carrier-free formulations in which all the particles are drug particles, the focus on how drug surface properties and cohesive behaviour can be optimised for DPI formulation performance is useful.

## 8. Expert Opinion and Final Remarks

The development of DPI formulations is still largely restricted to trial-and-error experimental approaches, due to the fact that the interactive effects of particle properties that influence the DPI formulation performance are complex and difficult to quantify. The capability of using measured particle properties in the prediction of the DPI formulation’s performance is slowly becoming a reality in combination with the development of new mixing technologies. Particularly, the novel isothermal dry particle coating, the device of interest in this research, potentially allows a reproducible scientific approach for the preparation of interactive mixtures for carrier-based DPI formulations [94]. With the bulk of the formulation being the carrier, there is a need to establish a relationship between a quantifiable carrier particle property and the DPI formulation performance. This will enable the use of QbD approaches to build quality into DPI formulations in the early formulation stages, rather than evaluating their quality through the resultant performance. The prevalent use of lactose as the carrier of choice in DPI formulations has resulted in extensive research on its modification to optimise DPI formulation performance. The effects of particle size, particle size fraction, shape, morphology and surface additives of lactose were investigated with useful discoveries. Through more research on the dynamics of particle interaction, cohesive and adhesive forces and their roles in the deagglomeration of composite particles, it is possible to relate quantifiable particle properties such as surface energy and other surface energy derivatives to DPI formulation performance. Using these values, a more direct and less ambiguous relationship has been established with DPI formulation performance. This presents the advantage of predictability along with optimisation over the conventional empirical methods that have been used to date. There is still a knowledge gap on how best to apply carrier particle modification methods to deliver predictable formulation performance through QbD. Although some work has been done using this approach, discoveries based on a robust experimental design are still lacking. This needs to be investigated, particularly for different grades of alpha lactose monohydrate to generate a better understanding of this widely used carrier.

## Figures and Tables

**Table 1:** Major literature reviews on optimisation of carrier-based DPI formulation performance.

Title of Article and Author(s)	Focus of Review Article
The Influence of Fine Excipient Particles on the Performance of Carrier-Based Dry Powder Inhalation Formulations	Active site and agglomerate theories to explain the effect of lactose fines
Jones and Price [48]	
Particle Engineering for Pulmonary Drug Delivery	Surface morphology and dispersion behaviour in relation to asperities
[49]	
Formulation strategy and use of excipients in pulmonary drug delivery	Hydrophobic lubricants Carrier free formulations
Pilcer and Amighi [5]	
Lactose as a carrier for inhalation products: Breathing new life into an old carrier	Impact of lactose physicochemical properties such as size, size distribution, shape and surface roughness of the particle, the presence of moisture, impurities or performance
Marriot and Frijlink [50]	
A critical view on lactose-based drug formulation and device studies for dry powder inhalation: Which are relevant and what interactions to expect	Carrier surface properties and role in drug–carrier interaction in relation to active sites on the carrier surface Role of amorphous spots and carrier fines in drug–carrier interactions The need for balance between cohesive and adhesive forces in DPI formulations and forces required for dispersion during inhalation and how this balance relies on the control of interparticulate force Relationship between active sites and high surface energy
de Boer, Chan [17]	
Physico-chemical aspects of lactose for inhalation	Physicochemical properties of lactose that affect DPI performance, including carrier size, distribution and shape; surface roughness; polymorphic form of the carrier; flow properties and electrostatic charge
Kou, Chan [25]	
Drug–lactose binding aspects in adhesive mixtures: Controlling performance in dry powder inhaler formulations by altering lactose carrier surfaces	Engineering of lactose surface morphology through surface smoothness, surface roughness, solvent-based coating and mechanical dry coating Characterisation of coating quality
Zhou and Morton [51]	
Technological and practical challenges of dry powder inhalers and formulations	Briefly mentions the use of crystalline and sieved lactose fraction as carriers in DPIs and passivation of active sites on carriers by ball milling, wet smoothing and use of FCAs
Hoppentocht, Hagedoorn [52]	
A proposed definition of the ‘activity’ of surface sites on lactose carriers for dry powder inhalation	Relationship between carrier surface activity and the energy with which they bind APIs Preferential occupation of active sites and retaining of drug particles on active sites during drug dispersion
Grasmeijer, Frijlink [53]	
Formulation Design of Dry Powders for Inhalation	Improving dispersion by lactose fines and FCAs; effects of lactose size, shape and morphology in dispersion Preferential attachment of lactose fines to high energy sites, which occurs as a result of clefts, amorphous domains, different crystalline orientations or other surface defects related to moisture, charge or contamination on lactose surface
Weers and Miller [54]	
From single excipients to dual excipient platforms in dry powder inhaler products	The use of lactose as a single excipient platform in DPI formulations and the introduction of a functional additive (Mg stearate used as a lubricant, FCA, stabiliser, water barrier and chemical stabiliser) to form a dual excipient platform for DPIs
Shur, Price [55]	
A review of factors affecting electrostatic charging of and adhesive mixtures for inhalation	General review on the impact of electrostatics on Dpi formulations Influence of polymorphic form and size distribution of lactose on electrostatic charge, and how surface charge on carrier and drug can affect the dry coating or mixing process and Dpi aerosolisation
Kaialy [56]	
Influence of physical properties of carrier on the performance of dry powder inhalers	Impact of carrier properties on aerosolisation including carrier particle size (size distribution), shape, morphology (surface roughness), density and geometric diameter. Role of fine carrier and associated theories (active site, agglomeration, fluidisation theory and buffer hypothesis)
Peng, Lin [3]	
Modelling the performance of carrier-based dry powder inhalation formulations: Where are we, and how to get there?	More general carrier-based review, focused on the key performance determinants of DPI formulations Carrier size and size distribution, concentration and size of fine carrier, carrier surface roughness and porosity and carrier shape were identified as carrier components that affect DPI performance
Elsayed and Shalash [57]	

**Table 2:** Key FPF-enhancing strategies.

DPI Enhancing Strategies	Rationale	References
Use of fine carrier particles as performance modulators	Active sites theory, Agglomerate theory	[26,58,59,60]
Carrier surface roughening	Carrier nanopores reduce adhesion force by reducing effective contact area between carrier and drug particles, while carrier micropores facilitate deagglomeration of fine drug particles	[71,72,73]
Carrier surface smoothening	Smoother surface increases surface contact area and reduces crevices where fine drug particles are tightly held to carrier particles	[60,71,74]
Use of force control agents	Passivation of active sites on carrier particles/reduction in cohesive forces through selection of FCAs, e.g., MgSt and Leucine	[68,70,75]
	Polymers, e.g., PVP and Ethyl cellulose	[24]
